# Spore Formation and Toxin Production in *Clostridium difficile* Biofilms

**DOI:** 10.1371/journal.pone.0087757

**Published:** 2014-01-30

**Authors:** Ekaterina G. Semenyuk, Michelle L. Laning, Jennifer Foley, Pehga F. Johnston, Katherine L. Knight, Dale N. Gerding, Adam Driks

**Affiliations:** 1 Department of Microbiology and Immunology, Loyola University Chicago, Stritch School of Medicine, Maywood, Illinois, United States of America; 2 Hines Veterans Affairs Hospital, Hines, Illinois, United States of America; University of Connecticut, United States of America

## Abstract

The ability to grow as a biofilm can facilitate survival of bacteria in the environment and promote infection. To better characterize biofilm formation in the pathogen *Clostridium difficile*, we established a colony biofilm culture method for this organism on a polycarbonate filter, and analyzed the matrix and the cells in biofilms from a variety of clinical isolates over several days of biofilm culture. We found that biofilms readily formed in all strains analyzed, and that spores were abundant within about 6 days. We also found that extracellular DNA (eDNA), polysaccharide and protein was readily detected in the matrix of all strains, including the major toxins A and/or B, in toxigenic strains. All the strains we analyzed formed spores. Apart from strains 630 and VPI10463, which sporulated in the biofilm at relatively low frequencies, the frequencies of biofilm sporulation varied between 46 and 65%, suggesting that variations in sporulation levels among strains is unlikely to be a major factor in variation in the severity of disease. Spores in biofilms also had reduced germination efficiency compared to spores obtained by a conventional sporulation protocol. Transmission electron microscopy revealed that in 3 day-old biofilms, the outermost structure of the spore is a lightly staining coat. However, after 6 days, material that resembles cell debris in the matrix surrounds the spore, and darkly staining granules are closely associated with the spores surface. In 14 day-old biofilms, relatively few spores are surrounded by the apparent cell debris, and the surface-associated granules are present at higher density at the coat surface. Finally, we showed that biofilm cells possess 100-fold greater resistance to the antibiotic metronidazole then do cells cultured in liquid media. Taken together, our data suggest that *C. difficile* cells and spores in biofilms have specialized properties that may facilitate infection.

## Introduction

Biofilms are organized communities of cells, often but not always on a surface (either natural or abiotic), encased in an extracellular matrix composed of polysaccharide, protein and/or extracellular DNA (eDNA), depending on the species and growth conditions [1]. Cells in the biofilm can differ from those grown in planktonic culture in physiology, gene expression and morphology [2], [3]. These differences, in combination with the specialized extracellular molecules may endow the biofilm with the ability to survive diverse environmental stresses. Most likely, in natural environments, the biofilm is the most common growth form [4]. Biofilm formation also appears to be common during bacterial infection. In fact, biofilms can be important to pathogenesis and the resulting disease. Relatively well understood examples include the roles of biofilms in recurrent urogenital infection caused by *Escherichia coli*, lung infections due to *Pseudomonas aeruginosa* in cystic fibrosis patients and chronic ear infections (otitis media) often caused by a variety of bacteria [5]–[7]. Biofilms facilitate infection by enhancing attachment to host tissue and resisting host defenses, including the immune system [8]. Biofilms also allow infecting bacteria to resist antimicrobial therapy. For example, in some cases bacteria in biofilms can be up to 1000 times more resistant to antibiotic treatment than the same cells growing planktonically [9]. As a result, some bacterial infections will recur even after repeated antibiotic treatment [10]. The mechanisms enabling biofilms to persist in the host and to evade antimicrobial therapies remain poorly understood.

A better understanding of biofilm formation by pathogens, and how biofilms contribute to disease and impact treatment could significantly improve disease prevention and treatment. In particular, it is important to identify the involvement of the biofilm lifestyle, if any, in diseases where it has yet to be clearly documented. A striking example of a pathogen where biofilms could play a major role in disease is *Clostridium difficile*. *Clostridium difficile* infection (CDI), is a leading cause of healthcare-associated (HCA) infection. CDI causes a spectrum of disease manifestations ranging from mild diarrhea to potentially lethal pseudomembranous colitis, toxic megacolon, shock, and death [11]. One recent study estimated the annual hospital cost of CDI to be $3.2 billion/yr [12]. It is widely acknowledged that improved therapies for CDI are urgently needed [13].

CDI initiates with ingestion of *C. difficile* spores, germination in the gastrointestinal tract, and then colonization of the large intestine [14]. Colonization requires depletion or impairment of the host microbiota, usually due to administration of antimicrobials, as is common in hospitals [15], [16]. The disease, whose symptoms range from mild diarrhea to severe and potentially life-threatening colonic inflammation, requires production of one or both of two major toxins: toxin A and toxin B [11], [17]. Both toxin A and toxin B are proinflammatory and cytotoxic, causing disruption of the actin cytoskeleton and impairment of tight junctions in human intestinal epithelial cells, with resulting fluid accumulation and extensive mucosal damage to the large intestine [17].

In recent years *C. difficile* strains producing a more severe disease have become prevalent in hospitals [18]. These epidemic (so-called hypervirulent) strains fall into several molecular typing groups designated as North American Pulse Field type NAP1, restriction endonuclease analysis group BI or PCR ribotype 027, collectively termed NAP1/BI/027. A major outstanding goal is the identification of the feature(s) of these strains that cause increased disease severity. Candidates include increased levels of the A and/or B toxins, an additional toxin (the binary toxin or CDT) and increased production of spores (although recent experiments argue against this last possibility) [19]–[21].

A major hurdle to effective treatment of CDI is that after apparently effective treatment, the disease will reappear in 20–30% of patients [22]. After this first recurrence, the disease will appear a second time in 33% or more of patients [23]. Worryingly, infection with NAP1/BI/027 and related binary toxin positive strains has been found to be a significant risk factor for increased recurrence, high mortality, and poor response to treatment [24], [25].

A role for biofilms in CDI has not been demonstrated. However, there is evidence suggesting that *C. difficile* forms communities in the host *in vivo* [26], [27], Semenyuk, E.G., Poroyko, V.A., Johnston, P.F., Knight, K.L., Gerding, D.N. and Driks, A., in preparation). Biofilm formation could play major roles in all phases of the disease and, especially, in recurrence, where the biofilm could enable cells to resist removal by the flow of luminal material in the GI tract, avoid the host immune system, and resist antimicrobials. Intriguingly, biofilms in *Bacillus subtilis* are sites of spore formation [28]. If this is true in *C. difficile*, then biofilms in CDI could participate both in persistence and in dissemination via the feces. Several lines of evidence are consistent with the hypothesis that CDI involves a biofilm. Biofilms (or biofilm-like growth) have been found in several Clostridium species including *C. perfringens*, *C. thermocellum* and *C. acetobutylicum* [29]–[31]. Furthermore, several recent reports document *C. difficile* growth in apparently organized communities on abiotic surfaces *in vitro* [32]–[34]. These studies show that the matrix possesses polysaccharide and protein that have not been characterized in detail, as well as eDNA. They also demonstrate that at least some level of sporulation occurs in the biofilm [32], [33], and that full biofilm formation requires the protein SleC [33] and the major post-exponential phase regulator Spo0A [32]. Consistent with other biofilms, *C. difficile* in a biofilm was shown to be resistant to the drug vancomycin, one of the major treatments for CDI [33].

These experiments establish several important features of the *C. difficile* biofilm *in vitro* and identify several key open questions: what are the levels of toxins in the biofilm? How does spore formation progress during biofilm formation and what are the distinguishing properties of spores in the biofilm, if any? How much variability is there in biofilm properties among the diverse clinically relevant strains? In this study, to address these questions and provide a more detailed description of the *C. difficile* biofilm, we established conditions for colony biofilm formation on polycarbonate filters, characterized the architecture of the resulting cellular communities by microscopy, and identified and partially characterized the classes of macromolecules in the biofilm matrix. We also compared the levels of toxin and spore production among clinical isolates. We then analyzed two properties of the resulting biofilm: suppression of germination and antibiotic resistance. Finally, we characterized these biofilms and, in particular, spores in the biofilms, by thin-section electron microscopy. Strikingly, we found that biofilm-associated spores are encased by a distinctive structure that we suggest is composed of components from dead cell debris.

## Materials and Methods

### Biofilm Cultivation

All *C. difficile* strains are human clinical isolates. We used the toxigenic strains BI6, BI17, J9, K14, VPI10463, 630 [19], [35], [36] and the low-toxigenic strain BYI (Gerding laboratory collection). Cells were cultured anaerobically (85% N_2_, 5% H_2_, and 10% CO_2_) in a Bactron IV chamber (Sheldon Manufacturing, Cornelius, OR) at 36°C.

To generate colony biofilms, we first produced overnight cultures by inoculating a culture in Tryptic Soy broth (BD Biosciences, Boston, MA) and incubating at 36°C until an OD 0.8 was reached. 1 ml of culture was then centrifuged and the pellet resuspended in 2 ml of Tryptic Soy broth. 8 µl of the resuspended culture was used to inoculate the center of a pre-sterilized black polycarbonate (PC) membrane (Millipore, Billerica, MA). The filter was then placed on solid Tryptic Soy medium and incubated at 36°C for 1–3 days or 5–6 days. For the purpose of spore production membranes were incubated for 14 days. Membranes were transferred to fresh media after 3 days, in cases when culture on solid medium was extended past that time.

### Electron Microscopy

To image biofilms by scanning electron microscopy, intact biofilms on filters were fixed in 4% glutaraldehyde in 10 mM phosphate buffer overnight and then in 1% osmium tetroxide in the same buffer for 1 hour. After dehydration in a graded ethanol series, specimens were transferred in 100% ethanol at 35°C to a Polaron E3100 (Polaron Instruments Inc, Hatfield, PA) for critical point drying. The samples were sputter coated with gold-paladium for 2 min in a Polaron E5100 Series II sputter coater. Specimens were viewed with a JEOL 840A SEM operating at 10 kV. To image biofilms by transmission electron microscopy intact biofilms on filters were placed in 4% glutaraldehyde in 10 mM phosphate buffer and left overnight at 4°C. They were then transferred into 1% osmium tetroxide in the same buffer and incubated overnight at 4°C. After this, they were prepared for thin-section electron microscopy as described [37].

### Confocal Laser-scanning Microscopy

To image cells in biofilms by confocal laser-scanning microscopy (CLSM), biofilms were stained with 0.05% calcofluor white in 1 M Tris, pH 9 (autoclaved to dissolve the reagent) for 15 min in the dark, followed by rinsing with deionized water. To minimize motion within the biofilm during preparation, biofilms were treated with 1% glutaraldehyde for 30 min prior to calcofluor staining. Samples were imaged with a LSM 510 microscope (Carl Zeiss Inc., NY, USA), using an excitation wavelength of 440 nm and emission of 500–520 nm. Images were analyzed using Zeiss LSM image examiner software.

### Lectin Staining

Concanavalin A conjugated to Texas red (Molecular Probes, Eugene, OR) was used to label extracellular polysaccharides in biofilms. Stock solution were prepared according manufacturer’s instructions and stored at −20°C in 100 µl aliquots. Concavalin A was diluted in 0.1 M sodium bicarbonate buffer before use, to a concentration of 100 µg/ml. To stain the biofilm, 100 µl of the lectin solution was carefully placed on the top of the biofilm while still on its membrane. After incubation for 1 hour at room temperature in the dark, excess staining solution was removed by washing four times with PBS (137 mM NaCl, 2.7 mM KCl, 10 mM Na_2_HPO_4_, 2 mM KH_2_PO_4_ ). To visualize individual cells, 1 µl/ml of deionized water SYTO 9 was added and kept in the dark for 5 min. Samples were imaged with a LSM 510 microscope (Carl Zeiss Inc., NY, USA), using an excitation wavelength of 488/561 nm and an emission wavelength of 561/615 nm.

### Analysis of Extracellular Matrix

We generated biofilm matrix extracts essentially as described [38]. In brief, after growth on membranes, biofilm material was scraped off and suspended in 0.5 ml of 0.9% NaCl. Biofilm bacteria were then pelleted by centrifugation (13000 rpm, 20 min, 4C) and the supernatant was filtered through a 0.22 µm PVDF membrane filter (Millipore, Billerica, MA). The filtered supernatant was incubated with 0.02% deoxycholate for 30 min at room temperature and then 1/10 volume of 100% trichloroacetic acid was added. After 2 hours on ice, the reaction was centrifuged by 14000 rpm for 10 min, and the resulting pellet was washed with cold acetone, resuspended in 4X protein loading buffer [39], and fractionated by 12% SDS-PAGE. To identify matrix proteins, bands from SDS-PAGE gels were excised and subject to tandem mass spectrometry (Alpalyse Inc., Palo Alto, CA).

To prepare protein extracts of cells harvested from biofilms, we took the cell pellet produced in the course of harvesting matrix (see above) and resuspended it in 0.5 ml of 0.9% NaCl. The cells were then centrifuged (5000 rpm, 20 min, 4°C) and the pellet washed twice with 0.9% NaCl. The resulting pellet was resuspended in 0.9% NaCl and sonicated (Branson 450 Sonifier, Danbury, CT) (output control 5) on ice in 1 min bursts for 5 min. The sonicate was centrifuged at 14000 rpm in a microcentrifuge for 30 min at 4°C. The supernatant was then removed, and the pellet resuspended in 100 µl 4× SDS-PAGE loading buffer and boiled for 5 min prior to electrophoresis. Surface protein extracts were prepared as described in [40]. Briefly, 0.04 volumes of 0.2 M glycine pH 2.2 were added to the cell pellet. After incubation for 30 min at room temperature, the suspension was centrifuged at 13000 rpm for 10 min and the supernatant neutralized with 2 M TrisBase. Proteins were visualized using Coomassie Brilliant Blue stain (Biorad, Hercules, CA).

### Antibiotic Susceptibility Testing

After inoculation of filters, biofilms were cultured for 20 hours and then transferred to Tryptic soy agar plates containing metronidazole (MP Biochemicals, Solon, OH, stock solution dissolved in DMSO) at 0, 1, 10 or 100 µg/ml. Each biofilm was sampled at 0, 6 and 24 hours. At each time point, the biofilm, still on its filter, was placed in 1 ml of BHI broth, mixed thoroughly, and then further diluted in BHI. Each serially diluted sample was plated on BHI agar by the drop plating method [41].

To measure metronidazole sensitivity, an overnight liquid culture at an OD between 0.9 and 1 was diluted 1∶20 with Tryptic soy broth and metronidazole was added to 0, 1, 10 or 100 µg/ml. Samples were taken at 6 and 24 hours, serially diluted with BHI and plated as described above. To normalize the changes in cfu, for each experiment we converted the number of viable bacteria to the log reduction in cfu. The values at each time point were averaged and the standard deviations were calculated.

### Sporulation and Germination Frequencies

We harvested spores from biofilms after 6 days of culture on a filter. The biofilm was resuspended in 0.5 ml of BHI (with 1% taurocholate when germination was induced). Sporulation on blood agar plates was performed as described [42]. After 6 days spores were scraped off the plates to 1 ml of PBS, spun down gently and resuspended in 0.5 ml of BHI with or without taurocholate. When spores were germinated, the culture was incubated anaerobically for 30 min and then analyzed by microscopy. Germinated spores were identified by observing the change in refractility that accompanies germination (phase bright to phase dark), using phase contrast microscopy. We performed three complete independent germination experiments. We used 700 to 1000 spores per experiment. In each experiment, we collected microscopy data in triplicate. Ten measurements were made for each replica. Data from all experiments were averaged. The standard error of the mean was calculated for each average.

### Toxin Analysis

To detect toxin in biofilm matrix extracts, we first measured the concentration of total protein in the matrix extract (using the BCA Protein Assay Kit, Pierce, Rockford, IL). For Western blot 5 µg of extract was fractionated on a 6% SDS-PA gel. After electrotransfer to a nitrocellulose membrane, we blocked the membrane overnight at 4°C in PBS with 5% nonfat milk and 0.1% Tween 20. The membrane was probed with a 1∶1000 dilution of mouse anti-toxin antibody obtained from *C. difficile* infected mice [43]. Bands were visualized by application of a secondary goat anti-mouse IgG conjugated to horse radish peroxidase (Jackson Immuno-Research, West Grove, PA) and the SuperSignal West Pico Chemiluminscent Substrate system (Pierce, Rockford, IL).

Total toxin (A and B) production was measured using the Wampole *C.difficile* TOX A/B II kit (Tech Labs Inc., Blacksburg, VA) according to the manufacturer instructions. EPS extracts containing 1 µg of total protein were diluted with the supplied diluent and analyzed in triplicates. Total toxin level was determined by measuring A_450_/OD_620_. We analyzed biofilms from three independent inoculations. Data was analyzed using one-way ANOVA and the two sample Student's T test (XLSTAT). P values <0.05 were considered statistically significant.

### Cytotoxin Assay

The assay was performed twice using Bartels Cytotoxicity Assay for *Clostridium difficile* Toxin (Trinity Biotech, Carlsbad, CA) as described by the manufacturer. Freshly prepared matrix extracts from 3 day-old biofilms were used after five-fold serial dilutions. Cell cultures were monitored on an inverted light microscope (20X magnification) at 24 and 48 hours. Cytotoxic titers were calculated as the reciprocal of the highest matrix extract dilution resulting in rounding of 100% of cells, divided by the µg of total soluble protein.

## Results

CDI involves colonization of the gastrointestinal tract [14]. We hypothesize that during CDI, *C. difficile* forms a biofilm. Therefore, to better understand the association between *C. difficile* and host tissue, we sought to culture *C. difficile* biofilms *in vitro*, by growing *C. difficile* on a surface under conditions that produce a multicellular community and characterizing the cellular organization of these communities, some of their properties, and the extracellular polymeric substances they produce.

### Characterization of Surface-cultured *C. difficile* Communities by Light and Electron Microscopy

We sought to identify static culture conditions that would result in formation of a surface-associated multicellular community encased in an extracellular polymeric substance (EPS). After attempting to culture *C. difficile* on a variety of abiotic surfaces, we found that growth was robust on the surface of a polycarbonate disk placed on an agar-medium plate [44]. To do this, we cultured *C. difficile* cells in liquid medium until mid-exponential phase and then used 8 µl of this culture to inoculate the polycarbonate disk. When applied to a variety of clinically relevant low-toxigenic (BY1), toxigenic (K14, VPI10463, 630 and J9) and BI/NAP1/027 strains (BI17 and BI6), this protocol resulted in the appearance of multicellular communities, that we refer to as colony biofilms from hereon based on the analysis described below. For each of these strains, we found that after 24 hours of incubation, a biofilm was readily visible on the disk surface and by 6 days, the biofilm was about 10–12 mm in diameter and 8–12 µm thick as measured by CLSM.

**Figure 1:**
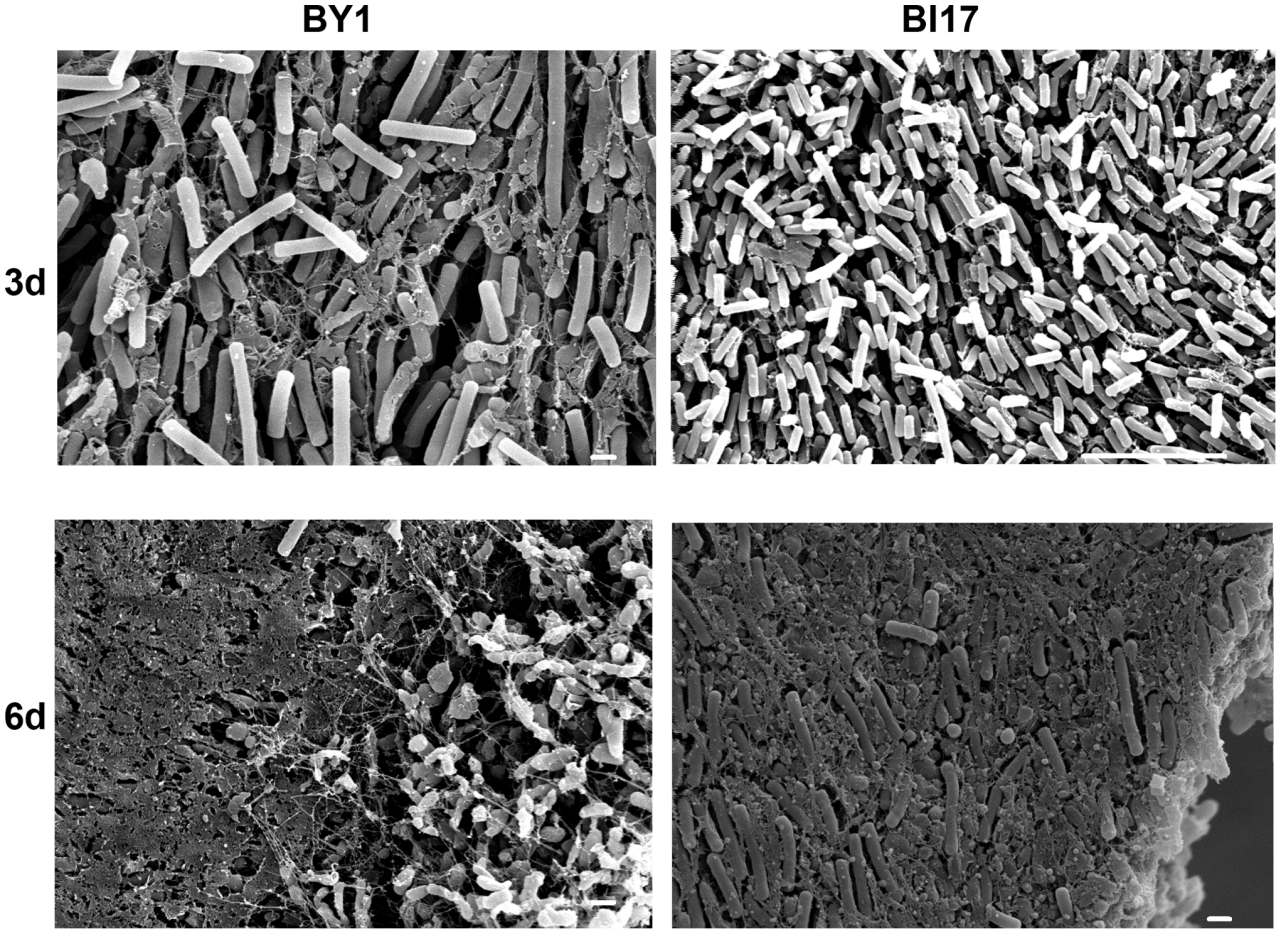
Scanning electron microscopic analysis of *C. difficile* biofilms. Biofilms were cultured for 3(upper panels) or 6 days (lower panels) from strains BY1 (left panels) and BI17 (right panels). Size bars indicate 1 (BY1-3, 6 days; BI17-6 days) or 10 µm (BI17-3 days).

To determine whether the *C. difficile* cellular communities on polycarbonate disks had the characteristics of a biofilm, we first imaged them by scanning electron microscopy (SEM). In this initial analysis, we imaged strains BI17 and BY1 at 3 and 6 days after inoculation of the polycarbonate filter. We regard 6 days of culture as the time needed to form a mature biofilm, for reasons discussed below. At both time points, we detected extensive mats of rod-shaped cells, interconnected by a network of extracellular material including pieces of cell debris and string-like material apparently connecting cells (Fig. 1). The appearance of this extracellular material is consistent with the possibilities that it is dead cell material or a secreted EPS. Overall, the appearance of these cellular colonies is consistent with their being biofilms (see, for example, [45]).

**Figure 2:**
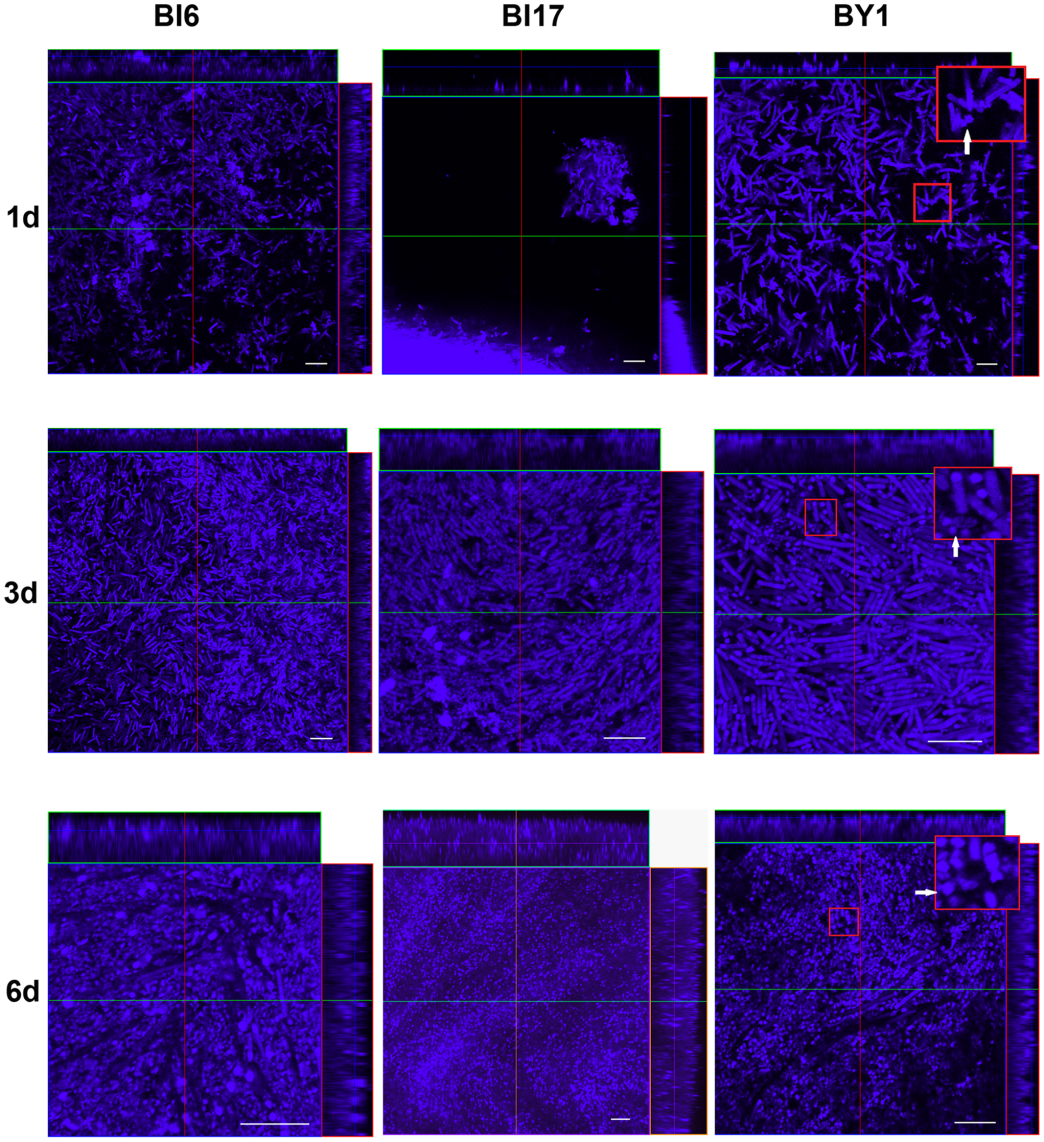
Confocal laser-scanning microscopic analysis of *C. difficile* biofilms. Biofilms were cultured for 1 (upper panels), 3 (middle panels) or 6 (lower panels) days from strains BI6 (left panels), BI17 (middle panels) and BY1 (right panels). In each panel, X–Y (center), X–Z (top), and Y–Z (right side) sections from a 3D data set are shown. At the upper right corner of each panel in the right column, there is an inset that is an enlargement of the boxed portion of the image. White arrowheads point to cell debris (uppermost panel) or spores (middle and lower panel). Magnifications in each panel have been chosen to assist in interpretation of the data (see the text). Size bars indicate 10 µm.

To more accurately describe the biofilm architecture and to visualize cells throughout the biofilm interior, we fixed the biofilms with glutaraldehyde, stained them with calcofluor-white, and then imaged them by confocal laser-scanning microscopy (CLSM). Calcofluor white binds to (1–3) and (1–4) beta- linked D-glucans and was previously shown to bind to the EPS in several bacterial biofilms [46]. We analyzed strains BI6, BI17, J9 and BY1 biofilms at 24 hours, 3 days and 6 days after inoculation of the polycarbonate filter. After 24 hours, we observed colonies of about 5–6 mm in diameter. CLSM revealed regions with a high concentration of apparently proliferating cells as well as smaller, punctate objects that we interpret as cell debris due to cell death (see below) (Fig. 2). Often, adjacent to these regions, we also observed smaller-sized regions harboring both cells and debris (Fig. 2). We also observed small colonies, some distance from the main biofilm colony, that contain vegetative cells as well as material we interpret as cell debris (Fig. 2). We interpret these colonies as sites of new growth, likely formed by cells that have migrated from the larger colony edge. We conclude from these data that after 24 hours, there is both cell growth and, most likely, cell death occurring in the colony biofilm.

After 3 days of culture, as at 24 hours, we observed both rod-shaped cells and apparent cell debris, suggesting that cell death continued along with cell division. To a large extent, cell division appeared to occur in chains, resulting in close packing of the cells (Fig. 2). We also detected the first appearance of shorter, ovoid cells in the biofilm (Fig. 2). Our initial hypothesis that these cells are spores was confirmed by parallel analysis of these cells using phase contrast microscopy, an imaging method that readily distinguishes bacterial spores from vegetative cells [47].

At 6 days, most of the cells in the biofilm had become spores (Fig. 2). We also observed a significant amount of apparent cell debris. In addition, however, we also detected isolated regions, within the colony, of vegetative cells that occupied a much smaller volume within the biofilm. These cells were clearly in the process of actively dividing. We did not observe spores or apparent cell debris in these regions. Also, as for 24 hour-old colonies, we observed small colonies containing apparent vegetative cells and cell debris outside the main colony (Fig. 2).

**Figure 3:**
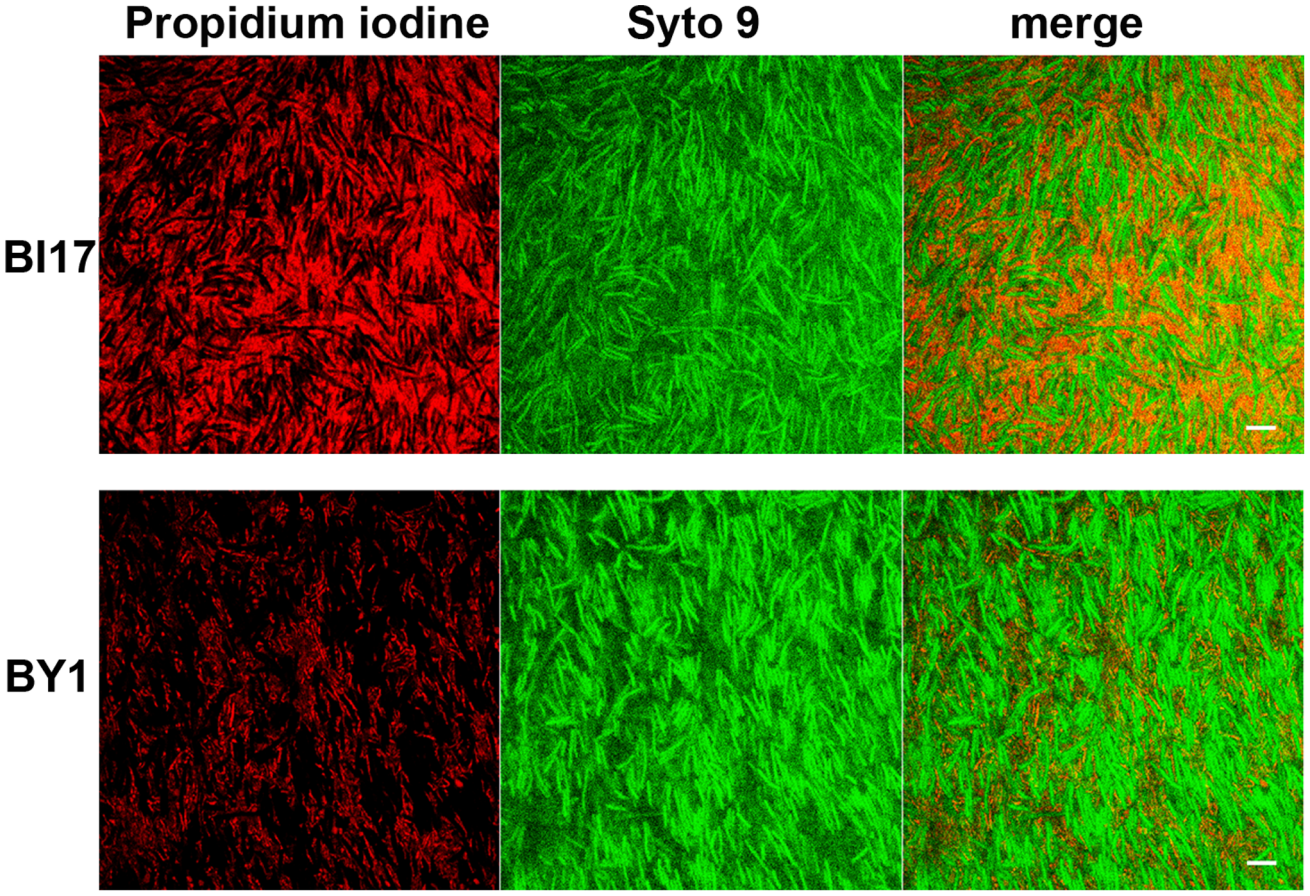
Confocal laser-scanning microscopic analysis of *C. difficile* biofilms stained with nucleic acid stains. 2-old biofilms from strains BI17 (top panels) and BY1 (bottom panels) were stained with propidium iodine (left panels) and Syto-9 (middle panels) and imaged so that the propidium iodine (left panels) or Syto-9 (middle panels) were visualized separately or together (right panels). Size bars indicate 10 µm.

The CLSM data are consistent with the possibility that regions between cells showing punctate fluorescence with calcofluor white staining contain dead cell debris. We also noticed cells in these regions that appeared relatively dark with Calcofluor staining, suggesting that these cells were dead (see, for example, Fig. 2). To address these possibilities, we stained 2 and 3 day old biofilms with a fluorescence stain cocktail designed to distinguish living and dead cells (Live Dead BacLight Kit (Invintrogen) (Fig. 3). The resulting data support the view that these regions contain dead cells and dead cell debris. Because the stains used in this experiment bind DNA, these data also argue that the regions between living cells are occupied by DNA. This interpretation is consistent with data presented previously [32], [33].

**Figure 4:**
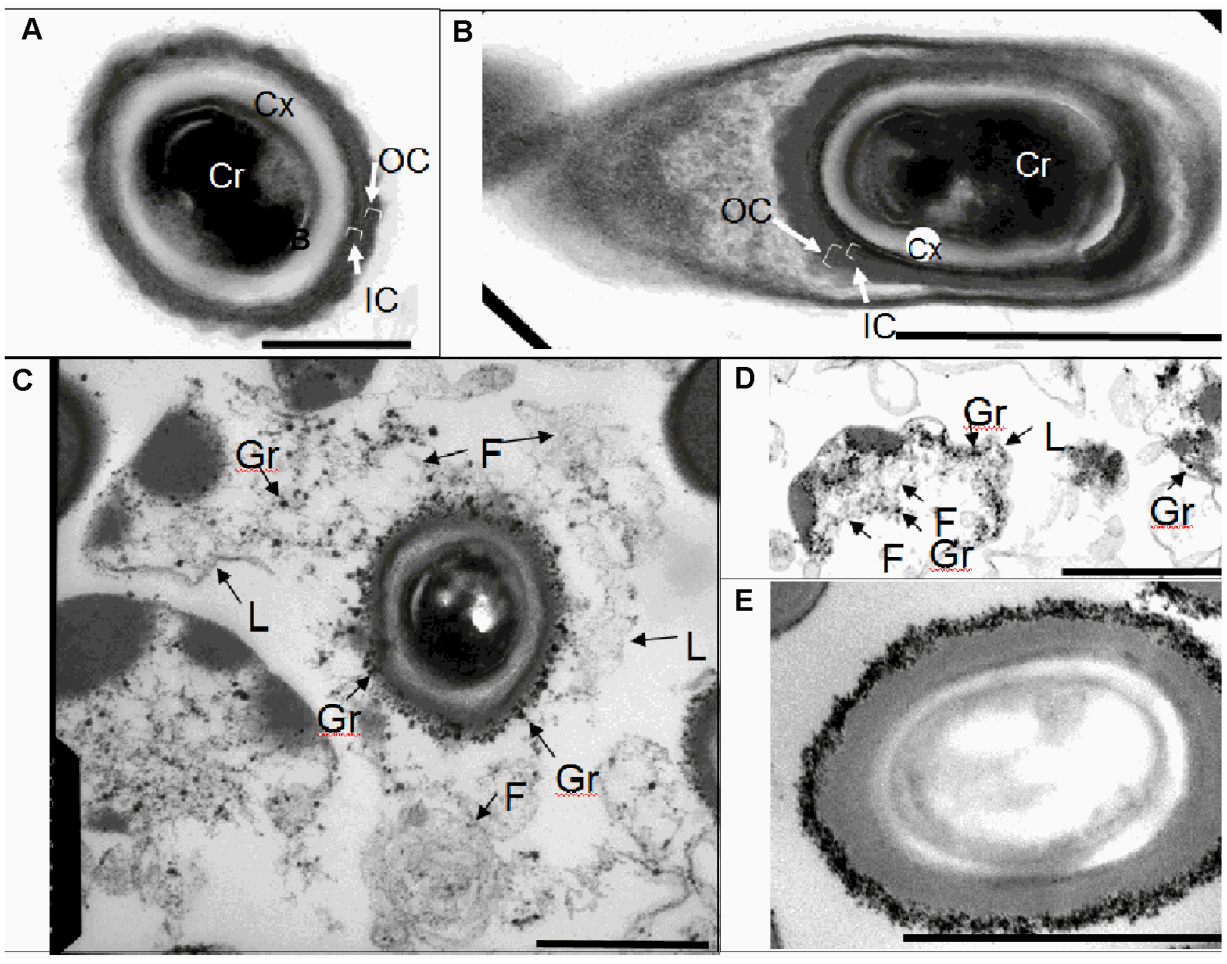
Transmission electron microscopic analysis of *C. difficile* spores in biofilms. A. Spore from a 3-old biofilm. B. Sporulating cell from a 3 day-old biofilm. C. Spore from a 6 day-old biofilm encased in a shroud. D. Matrix from a 6 day-old biofilm. In A, the outer coat (OC), inner coat (IC), cortex (the spore peptidoglycan, Cx) and core (the location of the spore DNA, Cr) are indicated. In B and C, granules (Gr), fiber-like structures (F) and a layer (L) are indicated. E. Spore from a 14 day-old biofilm covered with a thick layer of granules. The granules appear to be in close association with the coat surface. Bars indicate 300 nm (A), 1 µm (B), 1.5 µm (C), 600 nm (D) and 1 µm (E).

As detailed above, light microscopic analysis indicated that the biofilms contained vegetative cells, dead cells and spores. To confirm these interpretations and, in particular, to characterize the spores in more detail, we fixed intact biofilms from strains BI17 and J9, and imaged them by thin-section transmission electron microscopy (TEM). At six days, when spores were readily detected by confocal microscopy throughout the biofilm (Fig. 2), TEM revealed spores bearing key features in common with previous reports [48]. Specifically, we detected the spore core, cortex and coat (Fig. 4A). The *C. difficile* coat has a distinctive morphology, with a series of fine electron-translucent lamellae (resembling the *B. subtilis* inner coat [49]) and an outer layer lacking lamellae which has a scalloped outer edge. We suggest that this outer layer is more appropriately designated the outer coat rather than the exosporium [50]. Structures defined as exosporia in many other *Bacillaceae* species, including *B. anthracis*, *B. cereus*, *B. odysseyi* and *B. megaterium*, are separated from the coat by a gap known as the interspace [51]. In contrast, novel outer spore layers in very close association with the coat are more properly designated as additional coat layers (as in a recent case in *B. subtilis* [52]).

In addition, and in contrast to previous observations, we found that in the majority of spores, the coat is encased in a large sac-like structure (Fig. 4C–E). This structure appears to be bounded on its outer surface by a discrete layer and, in its interior, to possess a series of fine fiber-like structures as well as a series of punctate structures we call granules. Granules are abundant at the coat surface, appearing to be in very close association with the outside of the coat. Most likely, granules are the same structures seen surrounding the spore in figure 7E of [53]. Taken as a whole, we refer to this outer structure (that is, the granules and the layer in Figure 4C) as the shroud. The striking resemblance of the fine fiber-like structures and granules to the material dispersed throughout the biofilm (see, for example, Fig. 4D) leads us to speculate that the shroud is, to a large degree, composed of dead cell material. We further speculate that the layer of material that appears to encase the shroud is a remnant of dead cell envelope. This interpretation implies that the shroud is assembled only after the spore is released from the mother cell envelope at the end of sporulation. Consistent with this, three day-old biofilms possess released spores bearing neither the shroud nor any other structures surrounding the coat (Fig. 4A). Additionally, sporangia bearing unreleased spores, which are frequent in the three day-old biofilm, never show any evidence of a shroud or its initial formation (Fig. 4B and data not shown). Taken together, we conclude that the shroud is not an exosporium (in particular because it is not assembled in the mother cell as other exosporia are [51], [54], [55]) and suggest a model in which the shroud is harvested from the debris in the milieu.

To analyze the fate of the shroud in mature biofilms, we analyzed 14 day-old biofilms by TEM. We frequently saw spores bearing shrouds as just described. However, we also observed spores lacking the outer sac-like structure and, instead bearing only granules at the coat surface (Fig. 4E ). In these cases, the granules were frequently present at higher density than coat-associated granules in shrouds from 6 day-old biofilms.

### Composition of the *C. difficile* Biofilm Matrix

**Figure 5:**
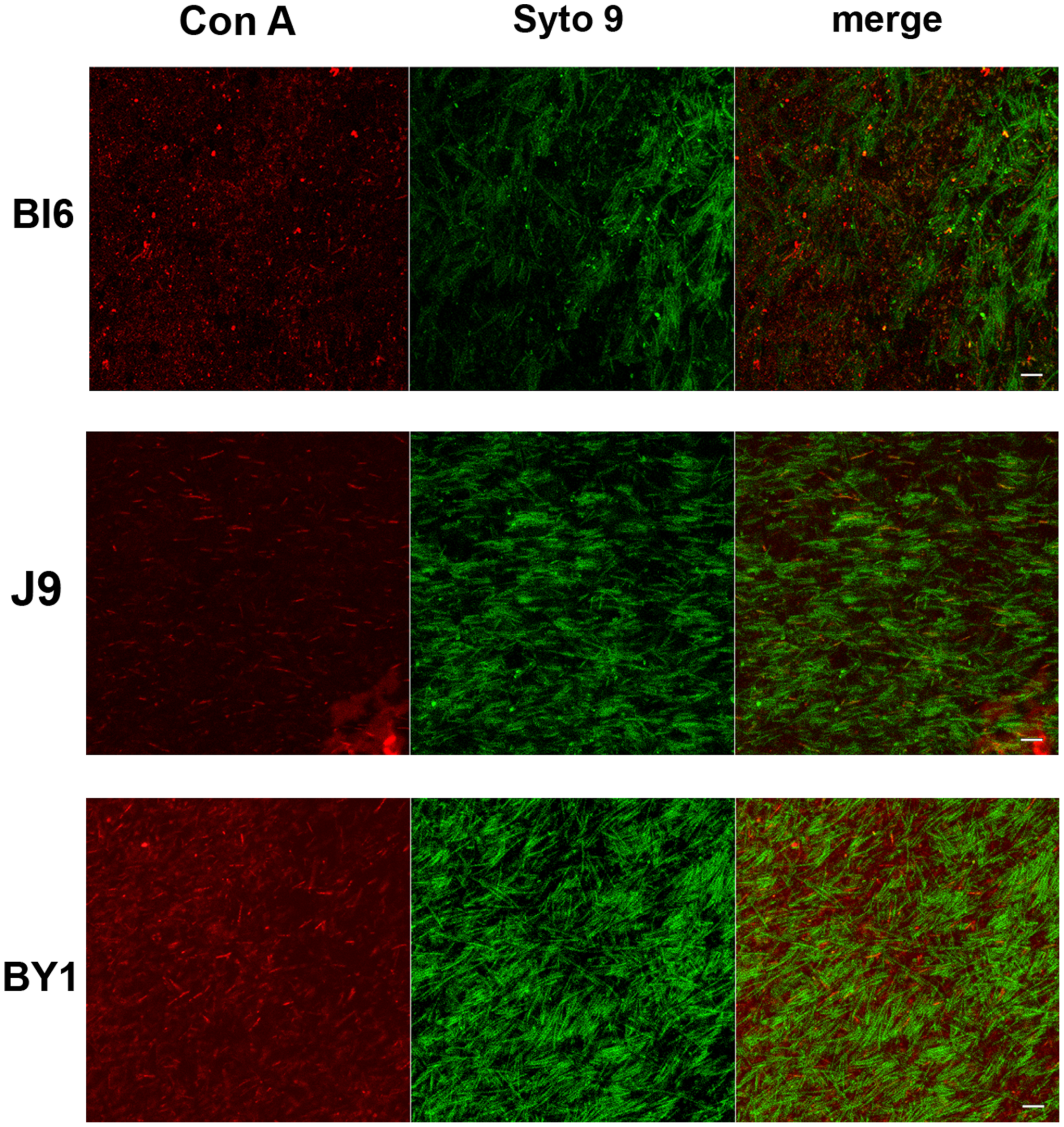
Confocal laser-scanning microscopic analysis of *C. difficile* biofilms stained with nucleic acid stains and concanavalin A-Texas Red. 3-old biofilms from strains BI6 (top panels), J9 (middle panels) and BY1 (bottom panels) stained with the lectin Concanavalin A and the nucleic acid stain Syto-9, and imaged so that the Concanavalin A (left panels) or Syto-9 (middle panels) were visualized separately or together (right panels). Size bars indicate 10 µm.

The biofilm matrix typically is composed of some combination of polysaccharide, protein, and nucleic acid [56]. Therefore, we searched for each of these classes of macromolecules in *C. difficile* biofilms. To detect polysaccharide, we applied polysaccharide stains to intact biofilms and localized the stains within the biofilms using CLSM. As already discussed, using the polysaccharide-binding stain calcofluor-white, in all strains we detected fluorescence around spores and vegetative cells as well as associated with material we interpret as dead cell debris (Fig. 2). From these data, we could not determine whether polysaccharide at these various locations was of one or multiple types. Since the types of polysaccharides in the biofilm, and their spatial distribution, could have functional significance, we applied fluorescently labeled concavalin A (ConA) to 1 and 3 day biofilms from strains BI6, J9 and BY1, and analyzed staining by CLSM. In contrast to staining with calcofluor white, ConA staining was limited to the regions between cells (Fig. 5). These data suggest that the polysaccharides in the intercellular regions, and the polysaccharides surrounding cells and spores, are not identical.

**Figure 6:**
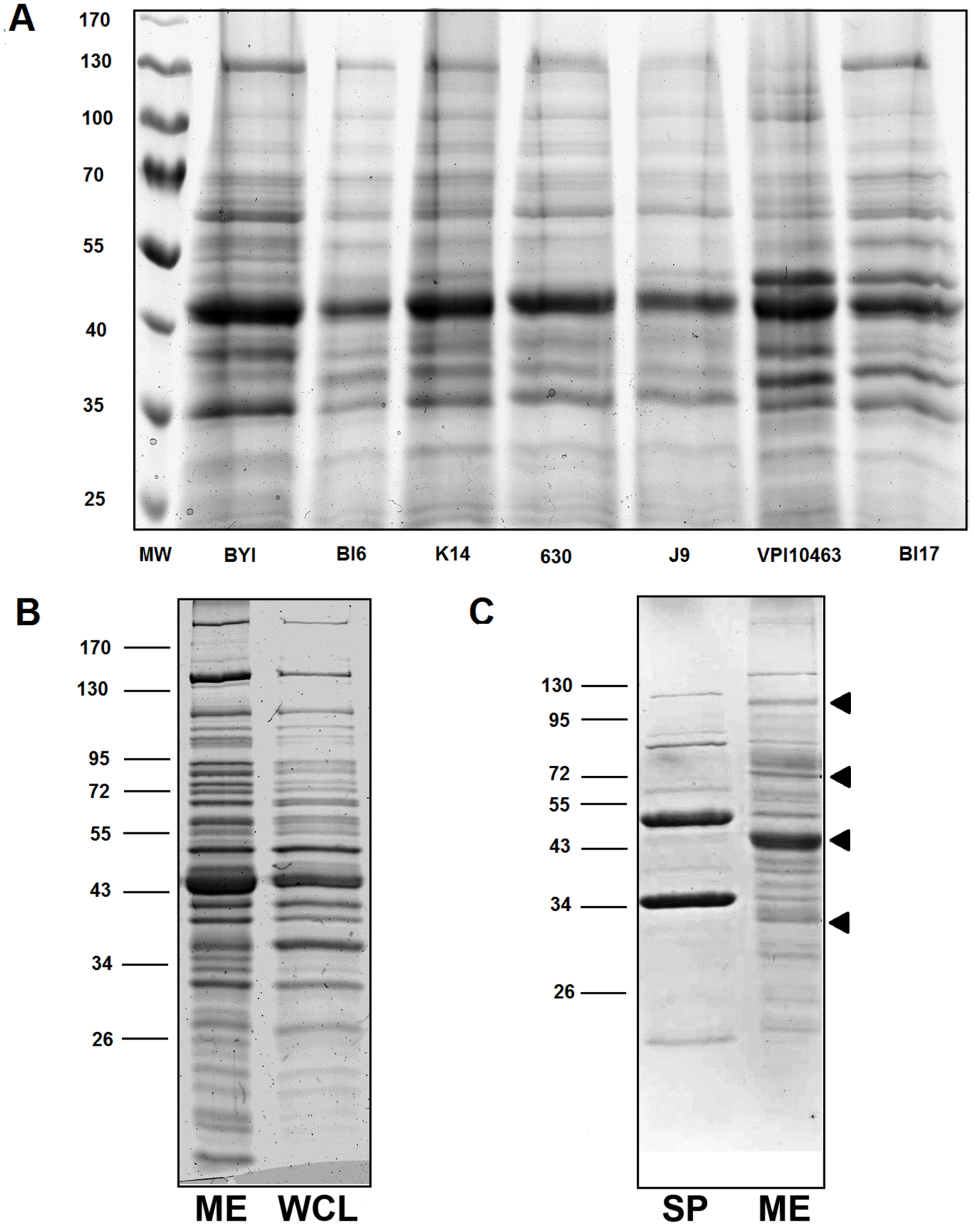
SDS-PAGE analysis of biofilm matrix extracts. A) Matrix was extracted from 6-day old biofilms of the strains indicated below each lane, electrophoresed on 12% PA gels and stained with Coomassie brilliant blue. Molecular weights are indicated in kD on the left. B) Matrix extracts (ME) or whole cell lysates (WCL) were analyzed from a 5-day old biofilm from strain BI17. C) An extract of surface proteins (SP) or matrix (ME) was analyzed from a 5-day old biofilm from strain J9. Arrows identify bands chosen for analysis by mass spectrometry.

To analyze the protein content of the matrix, we prepared biofilm extracts, fractionated them by SDS-PAGE and visualized the proteins by Coomassie Brilliant Blue staining. We detected a similar electrophoretic profile in all the strains (Fig. 6A). Proteins can potentially reach the matrix by a variety of mechanisms, including secretion, removal from the cell surface, lysis of cells, or some combination of these mechanisms. To distinguish among these mechanisms, we prepared cell-surface extracts, as well as extracts of whole cells and analyzed them by SDS-PAGE. From cell surface extracts, as expected, we detected prominent bands at 36, 53 and 70 kD, likely corresponding to known *C. difficile* cell wall-associated proteins [40] (Fig. 6C). Importantly, this electrophoretic pattern is dissimilar to that of the *C. difficile* biofilm matrix extract, indicating that matrix proteins did not arise from the cell surface. In contrast, the pattern produced from whole cell extracts was indistinguishable from the matrix extracts (Fig. 6B). The simplest interpretation of these data is that, to a large degree, the protein contents of the matrix is the result of cell lysis during biofilm formation.

**Figure 7:**
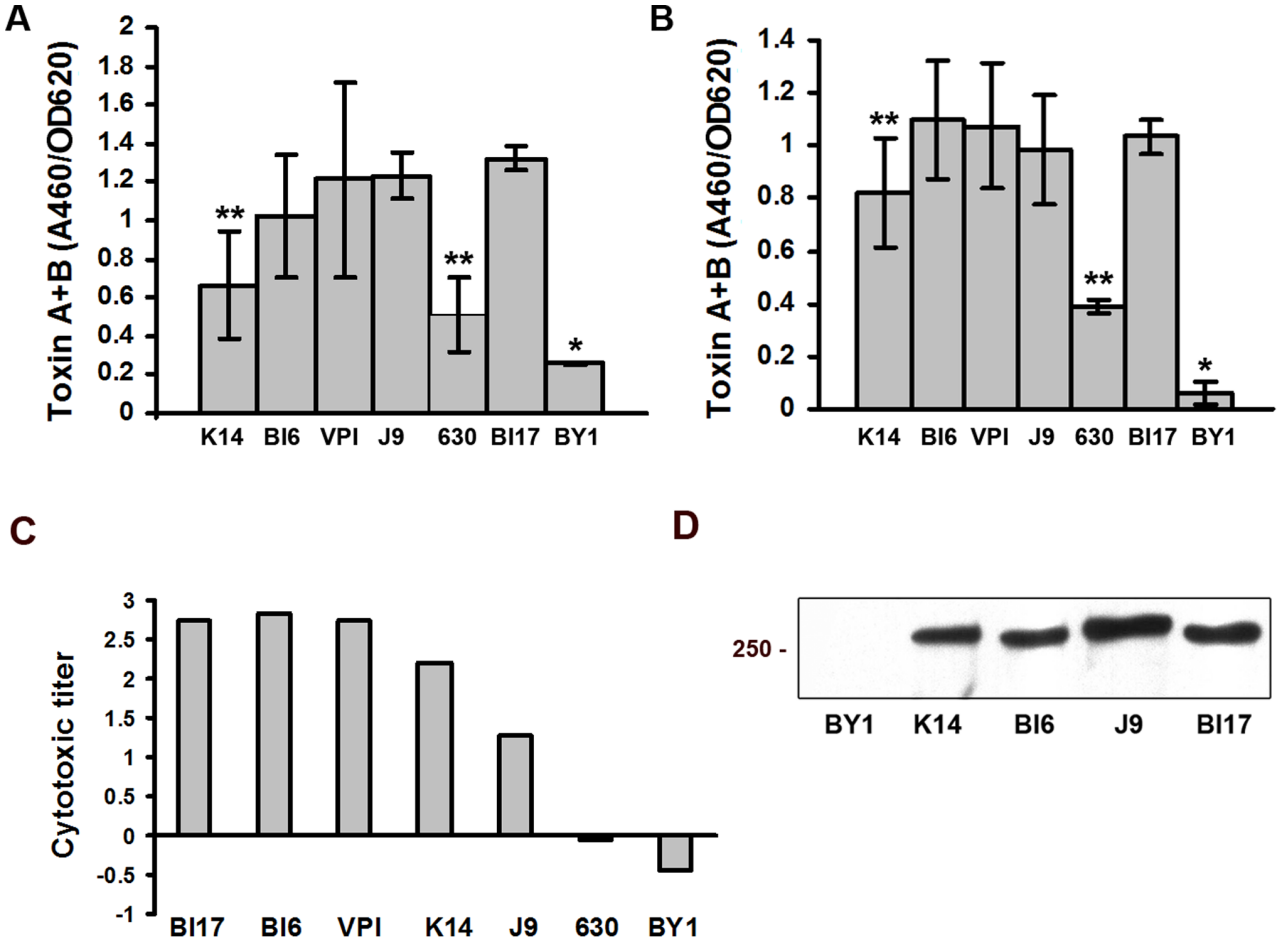
ELISA, western blot and cytotoxicity analysis of toxin A and B production in biofilms. A) Toxin A and B levels from the matrix of 3-day old biofilms were measured by the Wampole *C. difficile* TOX A/B II kit ELISA kit. *indicates that toxin levels differ significantly between BY1 and all other strains. **indicates that strains 630 and K14 differ significantly from all other toxigenic strains. The two sample t test (P<0.05) was used along with the ANOVA test. B) Toxin A and B levels from the matrix of 6-day old biofilms were measured by the Wampole *C. difficile* TOX A/B II kit ELISA kit. *indicates that toxin levels differ significantly between BY1 and all other strains. **indicates that toxin levels differ significantly between strain K14 and 630, and strains BI6, VPI10463, BI17. C) Cytotoxicity was assayed on 3 days old biofilms, using the Bartels Cytotoxicity Assay for *Clostridium difficile* Toxin. Cytotoxic titers are shown as the log of the reciprocal of the highest matrix extract dilution resulting in rounding of 100% of cells, divided by the µg of total soluble protein. The assay was performed twice with similar results. A representative experiment is shown. D) Western blot analysis of matrix extracts of 6-day old biofilms, using an anti-toxin A and B antibody. Matrix extracts were prepared from the strains indicated below each lane. A molecular weight standard is indicated on the left.

It is possible that proteins abundant in the matrix have important roles in biofilm function. While addressing this question in detail is beyond the scope of the present study, as a first step, we identified proteins in four of the most intensely staining bands using mass spectrometry. We specifically avoided bands that co-migrated with likely known cell wall proteins. We identified 7 proteins in these 4 bands. Strikingly, all are annotated as metabolic enzymes: formate-tetrahydrofolate ligase, acetyl-CoA acetyltransferase, 2-hydroxyisocaproate CoA-transferase, NAD-specific glutamate dehydrogenase, 3-hydroxybutyryl-CoA dehydrogenase, fructose-bisphosphate aldolase and formate-tetrahydrofolate ligase. While it is possible that these proteins contribute in some way to biofilm function, our data do not allow us to make any conclusions regarding the functions of these proteins in the matrix.

### Toxin Accumulation in the Biofilm

We hypothesized that *C. difficile* biofilms would produce and accumulate toxins A and B, the major known *C. difficile* virulence factors [11], [14]. To address this, we prepared matrix extracts and used ELISA to measure the combined levels of toxin A and B. Toxin was readily detected in all the toxigenic strains we tested (Fig. 7). In at least some experiments, we first detected toxin in biofilms after 24 hours (using western blot analysis, data not shown). However, the level was low and, in some experiments, toxin was not detected at this early time. We detected toxin consistently in 3 day-old biofilms, indicating that toxin appears at least by the time the biofilm is established (Fig. 7A). We also detected toxin consistently in 6-day old biofilms indicating that toxin remains detectable in the mature biofilm. The analysis of variance revealed a significant difference in toxin production at 3 and 6 day-old biofilm samples (F(5,34) = 9.08, P<0.05). This result allowed us to use the pairwise Student's T-test to analyze toxin production by the various strains. In 3 and 6 day-old biofilms, the difference in total toxin levels between strain 630 and any of the other toxigenic strains was significant (using the Student's T-test P<0.05) (Fig. 7B). The level of toxin in biofilms from stain K14 was slightly lower than in the other toxigenic strains at days 3 and 6. Overall, while the absolute levels of toxin varied among strains from experiment to experiment, the relative levels of toxin among strains was similar from experiment to experiment.

As an additional way to detect toxin in the *C. difficile* biofilm, we fractionated matrix extracts from 6-day old biofilms using 6% SDS PAGE and performed western blot analysis with antibodies that react against both toxin A and B [43]. We detected several bands between 170 and 250 kD in the toxigenic strains K14, J9, BI17 and BI6 but not in strain BY1 (Fig. 7D). Loading 10 times more of total soluble protein per line allowed us to see a weak but positive band in extracts of strain BY1 (data not shown).

The results of the western blot analysis were confirmed by the cytotoxicity assay. Strains BI17, BI6 and VPI showed the highest cytotoxicity titers (Fig. 7C). Strains K14 and J9 had intermediate cytotoxicity. We noticed some cell rounding in strain BY1 (which produces the lowest level of toxin), while strain 630 showed relatively low cytotoxicity, although higher than strain BY1.

### Efficiencies of Spore Production and Germination in Biofilms

**Figure 8:**
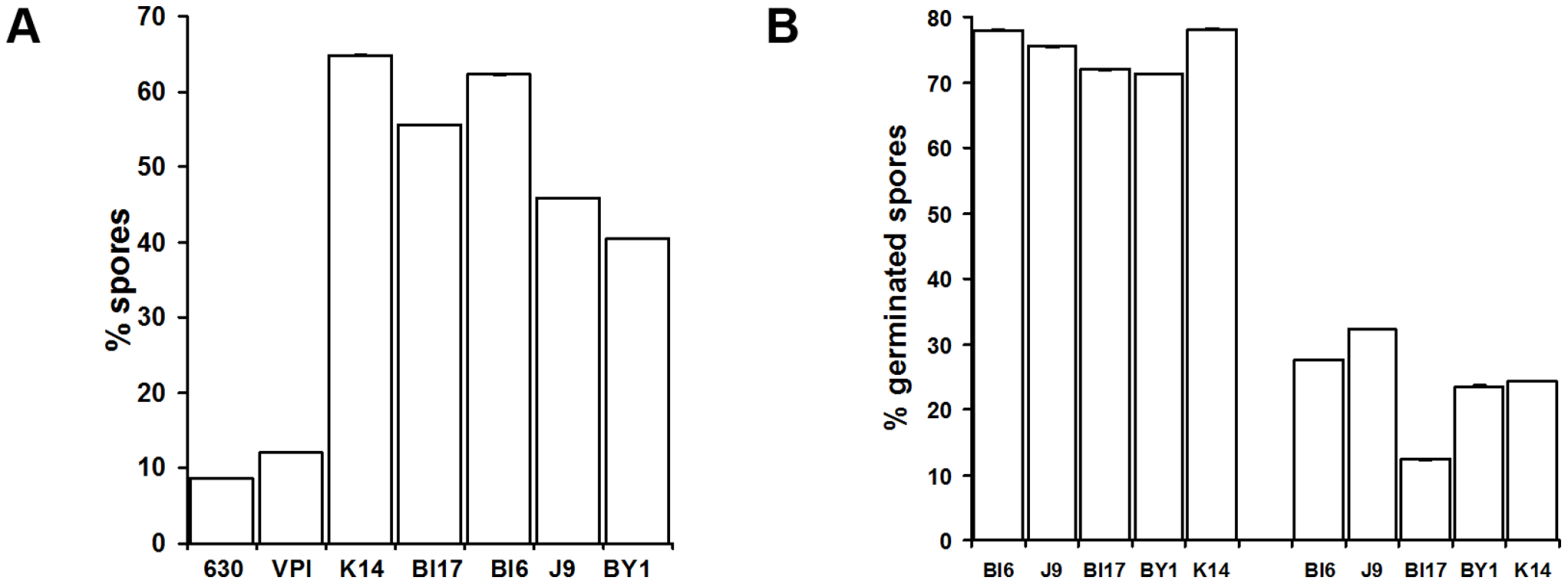
Spore production and germination in *C. difficile* biofilms. **A)** For each strain, cells were harvested from 6-day old biofilms and the numbers of spores and vegetative cells counted using phase-contrast light microscopy. Spore % was calculated as the # of spores/# of spores+# of vegetative cells. **B)** For each strain, spores were harvested from 6-day old cultures on Columbia blood agar plates or biofilms on T soy agar plates, treated with 1% taurocholate, and the numbers of germinated and ungerminated spores counted using phase-contrast light microscopy. Percentage of germinated spores was calculated as # germinated spores/# germinated spores+# ungerminated spores.

Sporulation and germination efficiencies could have significant impact on CDI. For example, it has been suggested that high sporulation efficiency could be a factor in the greater severity of disease caused by NAP1/BI/027 strains [25]. To address this, we measured sporulation efficiencies in biofilms by comparing sporulation levels in 6-day old biofilms with sporulation levels in on Columbia agar plates. Strains 630 and VPI10463, which are not usually associated with significant human disease, sporulated at 9 and 12%, respectively, which are relatively low levels and are consistent with previous observations [19]. However, the clinically isolated strains BI6, BI17, J9 and K14 had sporulation efficiencies that were relatively similar (between 46 and 65% (Fig. 8A). Importantly, there were no obvious differences in sporulation efficiencies in the biofilm between NAP1/BI/027 strains and non-NAP1/BI/027 strains. These data are consistent with the emerging view that variation in sporulation levels among strains are not a major contributor to any increase in disease severity between these recently epidemic strains and other *C. difficile* strains [21].

We hypothesized that biofilms could suppress germination and, thereby, facilitate the maintenance of a dormant population poised to cause recurrent disease [14]. To test this possibility, we measured the levels of germination of spores harvested from 6-day old biofilms and from sporulation on conventional, blood agar medium plates. Spores were harvested and, without washing (to retain potential spore-associated matrix components) then germinated by incubation in BHI medium supplemented with 1% taurocholate. To distinguish germination from outgrowth, we measured germination levels by using light microscopy to count phase bright spores. We found germination levels in conventionally cultured spores to be between 71 and 78% (Fig. 8B). In contrast, germination levels in biofilms were between 12–32%. Extended periods of germination did not result in significant increases in germination levels (data not shown). This is consistent with the view that spores in the biofilm have reduced germination efficiency.

### Impact of Biofilm Culture on Antibiotic Resistance

Antibiotic resistance is a well documented property of biofilms [9]. To determine whether *C. difficile* cells have greater antibiotic resistance when cultured under biofilm-forming conditions, we compared resistance to metronidazole treatment of biofilm cells from strains BI17 and J9, that were 20 hours old (and, therefore, had few or no spores and a lower density of cells) with cells grown in liquid culture. Metronidazole is one of the most commonly prescribed drugs for treating CDI [57]. We measured cell survival by CFU count.

**Figure 9:**
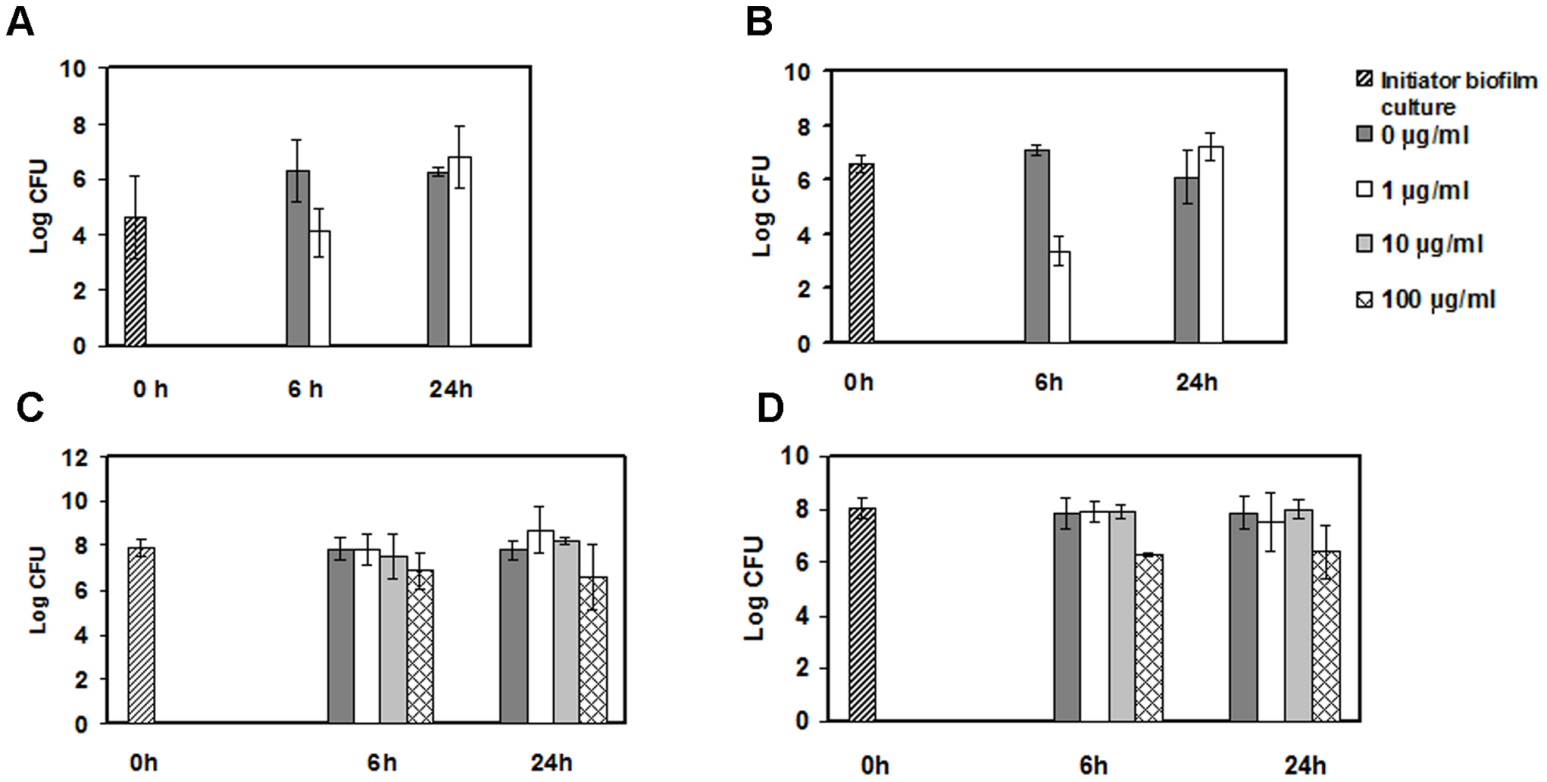
Effects of metronidazole on cell growth in biofilms. Effects of increasing concentrations of metronidazole on cells of strains J9 (A and C) and BI17 (B and D) grown in liquid culture (A and B) after 6 and 24 hours, or as biofilms (C and D). Biofilms were cultured for 20 hours before transfer to metronidazole. The data are from three experiments and presented as mean cfus.

In absence of metronidazole the number of cells in biofilms did not change significantly. Most likely, this is because of the simultaneous cell growth and death that takes place in the *C. difficile* biofilm (see, for example Fig. 2). In the absence of antibiotics, liquid cultures showed exponential growth as expected. After 6 hours, 1 µg/ml of metronidazole inhibited liquid cell growth by about 100-fold, and higher levels of metronidazole inhibited growth completely (Fig. 9A,C). The inhibition is consistent with previous findings [57]. Strikingly, in biofilm-cultured cells, we did not detect any growth inhibition at metronidazole levels of up to 10 µg/ml and only about a 10-fold reduction at 100 µg/ml (Fig. 9B,D). These data show that biofilms confer a 100-fold increase in resistance to metronidazole compared to cells growing in liquid culture.

## Discussion

There are three major conclusions from this study. First, we show that culture on a polycarbonate filter results in a biofilm whose architecture and cell type-distribution resembles that of *B. subtilis* and which possesses a matrix consisting of eDNA, polysaccharide and protein. These results are consistent with recent findings [33]. Second, we characterize sporulation in the *C. difficile* biofilm. These results identify a novel spore-surface structure that we designate the shroud. Third, we characterize properties of the biofilm relevant to CDI, including suppression of germination. We speculate that *C. difficile* biofilms might form in a variety of environments and, in particular, in the gastrointestinal tract during disease. We do not believe that any *in vitro* biofilm model will possess all the complex characteristics of this hypothetic host-associated biofilm. Nonetheless, we propose that *in vitro* biofilm growth conditions are an appropriate preliminary model for *C. difficile* growth, and especially sporulation, in a host. Important questions relating to potential biofilm formation during infection, such as the levels of sporulation or toxin production among clinical strains, can be profitably analyzed using the *in vitro* systems established here and by other researchers [32], [33].

A notable feature of the *C. difficile* biofilm is that, as in *B. subtilis* and *B. anthracis*, the biofilm supports abundant sporulation [28]. As visualized by CLSM, the general progression of events during sporulation appears similar between the *C. difficile* and *B. subtilis* biofilms. Notably, cell death at early stages of biofilm formation is common to both species. Our data suggest that in *C. difficile*, in addition to a likely role in supporting sporulation, cell death also facilitates delivery of toxin into the matrix. Cell death in biofilms is not restricted to sporulating species; is a common feature of biofilms in diverse species including *Pseudomonas aeruginosa*, where cell lysis occurs in localized regions inside microcolonies [58]. Given these observations *in vitro*, a key goal for the future is characterizing cell death and sporulation in *C. difficile* communities during infection. It is interesting that Ethapa et al. found only a low level of sporulation when biofilms were cultured on a plastic surface in BHI medium [33]. Other authors detected higher levels of sporulation, specifically in comparing strain R20291 to 630 [30]. This may reflect an effect of the growth surface and/or medium on developmental events during biofilm formation. We also note that Ethapa et al. observed a significant variation in biofilm biomass between a NAP1/BI/027 strain (R20291) and a non- NAP1/BI/027 strain (630) [33]. While we found that some biofilm features varied among clinical strains, we did not find that these differences were consistent in NAP1/BI/027 strains. In contrast to Ethapa et al. and Dawson et. al, [32], [33] we did not measure biomass. These variations in results suggest that the biofilm cultivation method has a significant impact on the resulting biofilm properties. Most likely, full characterization of *C. difficile* biofilms will require analyses using multiple culture systems.

Our studies indicate that biofilm matrix composition is complex. We do not yet know the roles of polysaccharide, protein or eDNA in biofilm formation, integrity or CDI. These molecules could have specialized roles in pathogenesis, including attachment to the mucosa and avoidance of host-defense. We suspect that matrix molecules contribute to germination inhibition and antibiotic resistance. We further speculate that the matrix plays a key role in CDI as a location where toxin can accumulate and, as a result, be directed to underlying epithelial cells, thereby facilitating host cell destruction during colonization. In spite of the likelihood that the matrix has roles in infection, the similarities between the biofilms of *C. difficile* and the non-infectious species *B. subtilis* suggests that at least some functions of matrix molecules may be best understood as supporting normal growth and/or sporulation rather than pathogenesis specifically [3], [59].

Using TEM, we identified a novel structure encasing the spore, the shroud, composed of fine fibers and darkly staining granules and, frequently, surrounded by a discrete thin layer. We interpret the shroud to be dead cell debris. Our observations suggest a model in which a spore acquires a shroud soon after mother cell lysis. We speculate that the ability of the spore to harvest dead cell debris is due to interactions between the granules and the coat surface. A further argument that the shroud is acquired only after spore release is that it is not present in the mother cell. We do not yet know the function of the shroud. It is plausibly involved in multiple spore properties including, possibly, germination inhibition, adhesion to other matrix molecules and the mucosa, and avoidance of immune recognition.

Taken together, our data argue that the shroud is not an exosporium. The exosporium is properly defined as a spore layer that is distinguished from the coat by not having a readily observed direct connection with the obvious coat layers that are in close apposition to the cortex [51], [60]. However, we suggest that an additional criterion for an exosporium is that it is formed in the mother cell, as is true for all other structures considered to be exosporia, so far (see, for example, [61]). The adhesion of material from the culture milieu to the spore surface is not unprecedented; vegetative cell-surface layer proteins adhere tightly to the *B. anthracis* spore surface [62]. Possibly, such adherence is adaptive in diverse environments.

The identification and characterization of the *C. difficile* biofilm presented here is useful to a better understanding of CDI. For example, our data argues that at least some important biofilm properties among clinical strains are very similar. Therefore, variation in biofilm properties among strains might not be a major cause of the increased disease severity associated with infection with NAP1/BI/027 strains. In particular, our data are consistent with the emerging view that disease severity does not correlate with sporulation levels [21]. Our data also have important possible implications for understanding recurrent CDI. At least 20% of patients successfully treated for CDI symptoms have a recurrence within 30 days [14]. In a significant number of cases, reinfection is with a strain that is genetically identical to that of the initial infection [63], suggesting that even after apparently successful treatment, *C. difficile* cells can remain in the patient. We hypothesize that biofilm formation plays a significant role in this ability to persist in the host. Specifically, the ability to form spores in the biofilm, and to suppress germination, could be major factors in recurring infection. Antibiotic resistance could result in the persistence of cells in biofilms in the host in spite of treatment.

Taken as a whole, we argue that studying the *C. difficile* biofilm in laboratory culture provides a much deeper understanding of the behavior of this organism in association with the host during pathogenesis and, at the same time, provide important insights into features of the biofilm lifestyle conserved among all spore-forming species.
